# Acute *Datura Stramonium* poisoning in East of Iran - a case series

**Published:** 2012

**Authors:** Mahnaz Amini, Hamid Khosrojerdi, Reza Afshari

**Affiliations:** 1 *Lung and Tuberculosis Research Centre, Mashhad University of Medical Sciences, Mashhad, I. R. Iran*; 2 *Department of Toxicology, Imam Reza Hospital, Mashhad University of Medical Sciences, Mashhad, I. R. Iran *

**Keywords:** Alkaloids, Atropine, Cholinergic Antagonists, *Datura Stramonium*, Poisoning

## Abstract

**Objectives**: *Datura Stramonium* (DS) is a common weed along roadsides, in cornfields and pastures and in waste areas. It belongs to the family *Solanaceae* and its toxic components are tropane belladonna alkaloids. It has been used voluntarily by teenagers for its hallucinogenic effect. The plant is named in Iran as Tatoore. Symptoms and signs of acute *D. Stramonium* poisoning usually are similar to anticholinergic syndrome. This study is done in order to clarify the status of this poisoning in our region.

**Materials and Methods:** This study is a case series on all patients admitted to Imam Reza Hospital, Mashhad, Iran, with acute *D. Stramonium* poisoning between 2008 and 2011. We observed their symptoms, signs, routine laboratory test results and treatment used to control their symptoms.

**Results:** There were 19 patients included in our study. Children were poisoned more commonly than teenagers and poisoning in adults was rare. All of the children ingested the plant accidentally. The most presenting symptom was irritability and the most common sign was sinus tachycardia. There was not any presentation of seizure or coma. Most of the symptoms were controlled by parenteral benzodiazepines and there were no need to use of cholinergic agents such as physostigmine.

**Conclusion:** Our study showed most of *D. Stramonium* poisoned population in our region are children. We suggest decreasing accessibility to the plant in order to decrease the incidence of its poisoning.

## Introduction

**Figure 1:**
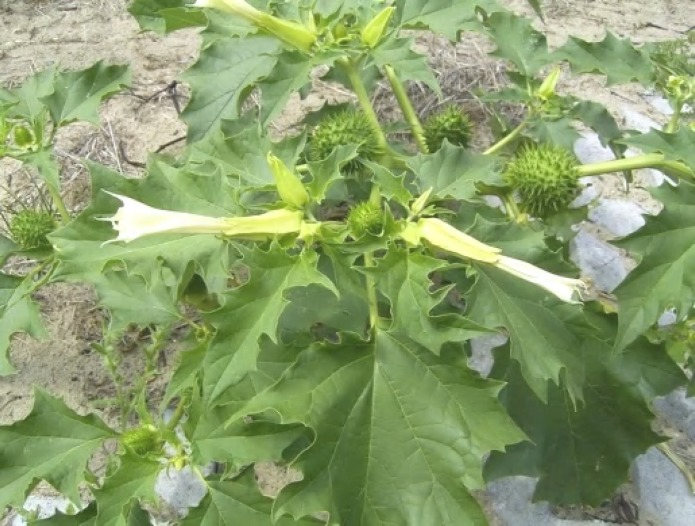
*Datura Stramonium* in blossoms

*Datura Stramonium* (*D. Stramonium*) is a weed from the family *Solanaceae*, the potato or nightshade family. The plant is native to Asia, but is also found in the West Indies, Canada, and the United States (Figure 1). 

The toxic principles are tropane belladonna alkaloids (Binev et al., 1995 ▶). It is known with different names across the world such as Angel’s trumpet, Jimson weed, Devil's trumpet, Devil's weed, Thorn apple, Jamestown weed, Stinkweed, Locoweed, *Datura*, Devil's cucumber and Hell's Bells. The clinical signs usually appear within 1 to 4 hour after ingestion (Tannis et al., 2006 ▶). However, the timing of ingestion is often hard to determine unless witnessed. As the plant contains anticholinergic alkaloids, its poisoning presents with symptoms and signs of anticholinergic syndrome. This syndrome results from the inhibition of central and peripheral muscarinic neurotransmission. 

Most cases of acute *D. Stramonium* poisoning occurred among teenagers after voluntary ingestion of the plant for its hallucinogenic and euphoric effects (Bouziri et al., 2011 ▶). The intoxication with *D. Stramonium* in horses was characterized by changes in some hematological indices such as erythrocytosis, increased hematocrit level, and low erythrocyte sedimentation rate (Binev et al., 2006 ▶). 

Sinus tachycardia is common and does not require treatment for a stable patient. Agitated or hallucinating patients often respond to reassurance and a darkened room. If chemical restraint is required, benzodiazepines are the drugs of choice. Tropane alkaloids are lipophilic and cross the blood-brain barrier; hemodialysis and hemoperfusion are generally ineffective. No effective methods exist for changing distribution or elimination of tropane alkaloids. Treatment largely includes control of anticholinergic toxicity usually by supportive measures. Activated charcoal is most useful in the first hour after ingestion. The decision to proceed with gastrointestinal decontamination should be individualized. 

Early gastric emptying should be considered for large ingestions of an initially asymptomatic patient or in an intubated patient regardless of the time of ingestion. Agitation should be controlled by titrating intravenous benzodiazepines to sedation. In most agitated patients, the risks of gastric lavage outweigh the potential benefits (Rivera et al., 2006 ▶). Specific antidote for tropane alkaloid toxicity is physostigmine salicylate, a reversible acetylcholinesterase inhibitor capable of directly antagonizing CNS manifestations of anticholinergic toxicity. Its role has been controversial in the management of *D. Stramonium* poisoning. This is due to its potential adverse effects secondary to acetylcholine accumulation (Tannis et al., 2008 ▶). These include seizures, muscle weakness, bradycardia, lacrimation, salivation, bronchorrhea, diarrhea, and asthma exacerbation (Rivera et al., 2006 ▶). Physostigmine can induce a life-threatening cholinergic crisis (e.g., seizures, respiratory depression, asystole). Therefore, it is usually reserved only for reversal of life-threatening complications of tropane alkaloid poisoning (e.g., tachydysrhythmias with hemodynamic compromise, seizures refractory to other therapeutic interventions, and severe agitation or hallucinations unresponsive to other therapy (Wagner et al., 2012 ▶).

## Material and Methods

We retrospectively studied the charts of all patients admitted with acute *Datura Stramonium* poisoning to Imam Reza Hospital from Aug 2008 to Aug 2011. Clinical symptoms and signs of patients with special attention to the presence of anticholinergic syndrome were assessed. We also looked for the results of laboratory tests (e.g., blood glucose, Serum urea, creatinine and electrolytes) conducted in the first hour of admission. Admission electrocardiograms were also assessed for conduction delays or arrhythmias. 

Subjects who were poisoned by more than one poisonous agent or those with abnormal test results were excluded from the study. Treatment options used for the control of symptoms were gathered from the charts. Data were analyzed using SPSS 11 software. 

## Results

**Figure 2:**
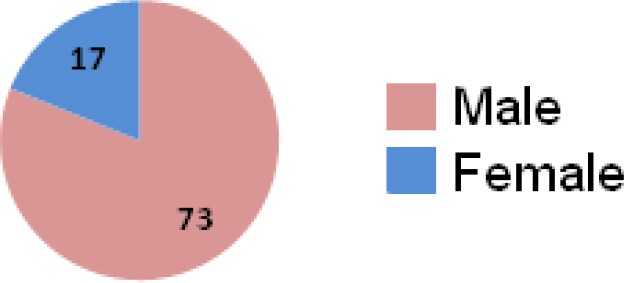
Incidence of acute Datura Stramonium poisoning by sex.

A total of nineteen patients with mean age of 12.2 years (3-29 yr) were included in the study. Fifteen patients were children who %67 of them were males (Figure 2). However, in adults acute *D. Stramonium* poisoning were occurred exclusively in men. 

The incidence of symptoms and signs in the first 6 hours in patients with acute *D. Stramonium* poisoning was as below: the most common finding was the constellation of anticholinergic syndrome. Sinus tachycardia, skin dryness, flushing, blurred vision and drowsiness were the most common symptoms and signs. The other symptoms and signs are ranked in (Table-1) Hallucination which is the usual purpose of misuse of *D. Stramonium* by different routs was reported in only 21% of our patients. The most dangerous symptoms of hyperpyrexia and ataxia were not common (15%) findings in our patients. None of our patients had serious CNS effects of the plant, such as seizure or coma.

**Table 1:** Incidence of symptoms and signs in acute intoxication with Datura Stramonium in order of frequency.

**Symptoms and Signs**	**No. (%)**
Sinus tachycardia	18 (94%)
Flushing	17 (89%)
Dry skin and mucosa	17 (89%)
Blurred vision/ photophobia	16 (83%)
Altered mental status	15 (78%)
Agitated delirium	14 (73%)
Disorientation	9 (47%)
Urinary retention	6 (31%)
Tremoulouness	6 (31%)
Decreased bowel sounds	5 (26%)
Hallucination	4 (21%)
Myoclonic jerks	4 (21%)
Hyperpyrexia	3 (15%)
Ataxia	3 (15%)
**Total**	**19 (100%)**

Electrocardiography of 94% of patients at presentation showed sinus tachycardia. Otherwise ECGs were normal. 

Treatment of intoxicated patients was mostly supportive. Sixteen out of nineteen patients were discharged from the hospital with total improvement of symptoms after use of a single dose of benzodiazepine in the first 24 hour of admission. None of our patients required the treatment with cholinergic agents such as physostigmine. 

## Discussion

Vanderhoff et al. Reported the teenagers as the most prevalent age group for poisoning with *Datura Stramonium* (Vanderhoff et al., 1992 ▶). Intentional misuse by teenagers who eat seeds, drink tea and or smoke cigarettes made of *D. Stramonium* has been reported by many authors (Coremans et al., 1995 ▶; Arouko et al., 2003 ▶; Wiebe et al., 2008 ▶). However, our study showed a different distribution of age. Acute DS poisoning in East of Iran is more common in children. It is nearly rare in adults. The change in pattern of senile distribution seems to be related to the route of poisoning in our region that is mostly accidental. *D. Stramonium* is not known for its euphoric effect between teenagers or young people in our region. Apart from the difference in the purpose of ingestion, the incidence of clinical symptoms and signs is similar to other articles in the literature. Our patients neither presented with seizure or deep coma nor showed these serious neurologic complications during their hospital stay. Treatment protocol in our study was using parenteral benzodiazepine with goal of mild sedation and it was effective as shown in the previous studies. None of our patients required physostigmine for the control of anticholinergic symptoms. Some of the cases in the literature ended up in death as the use of *D. Stramonium*. In our study there were no reports of short term mortality during this 3 year survey. 

It seems necessary to confirm this data by a prospective cohort with specific attention to the dose of ingestion and its relation to the type and duration of symptoms or signs.
